# Impact of the national health insurance coverage policy on mecapegfilgrastim utilization for chemoradiotherapy-induced neutropenia in cancer patients in China: a retrospective real-world analysis

**DOI:** 10.3389/fphar.2025.1546261

**Published:** 2025-02-17

**Authors:** Wenqing Fang, Jizhong He, Ying Wang, Yulei Zhu, Xiaodan Qian, Dan Su, Jinhong Gong, Jingjing Shang, Yuan He, Hong Wu, Xin Li

**Affiliations:** ^1^ Department of Medical Ethics Supervision, The First Affiliated Hospital of Soochow University, Suzhou, China; ^2^ Institute of Medical Humanities, Nanjing Medical University, Nanjing, China; ^3^ School of Marxism, Nanjing Medical University, Nanjing, China; ^4^ Department of Infection Management, The First Affiliated Hospital of Soochow University, Suzhou, China; ^5^ Office of Scientific Research, The Affiliated Stomatological Hospital of Nanjing Medical University, Nanjing, China; ^6^ Department of Pharmacy, The Second People’s Hospital of Changzhou, The Third Affiliated Hospital of Nanjing Medical University, Changzhou, Jiangsu, China; ^7^ Laboratory for Digital Intelligence and Health Governance, Nanjing Medical University, Nanjing, China; ^8^ Department of Pharmacy, The Second People’s Hospital of Lianyungang, Lianyungang, China; ^9^ Department of Health Policy, School of Health Policy and Management, Nanjing Medical University, Nanjing, China; ^10^ Department of Clinical Pharmacy, School of Pharmacy, Nanjing Medical University, Nanjing, China; ^11^ Center for Global Health, School of Public Health, Nanjing Medical University, Nanjing, China

**Keywords:** mecapegfilgrastim, national health insurance coverage, adjuvant chemoradiotherapy, utilization, binary logistic regression

## Abstract

**Objective:**

This study aimed to evaluate mecapegfilgrastim utilization for the prophylaxis of chemotherapy-induced neutropenia in cancer patients and to assess changes caused by the National Health Insurance Coverage (NHIC) policy.

**Methods:**

Individual patient data, including demographics, medical insurance status, cancer type, and tumor stage, were extracted from electronic medical records in an oncology specialty tertiary hospital in Jiangsu Province, China. An interrupted time series (ITS) analysis with a segmented regression model was applied to evaluate the NHIC policy’s effects, and multivariate binary logistic regression analysis was used to identify key factors influencing mecapegfilgrastim utilization.

**Results:**

The proportion of cancer patients receiving mecapegfilgrastim increased from 8.17% before the NHIC policy implementation to 36.05% after its implementation (P < 0.001). Utilization rose abruptly following the policy intervention (β = 0.143, P < 0.001) and continued to increase significantly afterward (β = 0.011, P = 0.004). However, inequities were observed in mecapegfilgrastim usage among patient subgroups, with utilization closely associated with patients’ location, cancer type, and tumor stage after the policy implementation.

**Conclusion:**

The NHIC policy significantly increased mecapegfilgrastim utilization, enabling more cancer patients to access this medication and effectively benefiting them. To address persistent inequities, the government should consider introducing additional measures, such as increasing the insurance reimbursement cap and separating the cost of expensive innovative anticancer medicines from hospital medical insurance budgets.

## 1 Introduction

Cancer has become the leading cause of death globally (Bray et al., 2021). According to the International Agency for Research on Cancer, there were 19.3 million new cancer cases and 10.0 million cancer-related deaths worldwide in 2020 (Sung et al., 2021). In recent years, due to the high morbidity and mortality associated with cancer, the global pharmaceutical industry has been actively pursuing novel anticancer therapies to meet the growing demand for oncological treatments. With advancements in medical technology, chemotherapy has emerged as a significant therapeutic approach for managing solid and hematologic tumors (Bonadonna, 1990). Additionally, chemotherapy plays a key role in both adjuvant and advanced cancer treatment settings. However, myelotoxicity often limits its clinical application, particularly in life-threatening conditions such as neutropenia and febrile neutropenia (FN) (Tirelli et al., 2020). FN increases the risk of infection and mortality and prolongs the intervals between chemotherapy cycles (Gu and Zhang, 2021). It is a common complication in cancer patients undergoing chemotherapy, frequently resulting in hospitalizations, chemotherapy delays, and potentially life-threatening outcomes (Gu and Zhang, 2021; Lyman et al., 2014; Ba et al., 2020). Therefore, immediate attention is essential for cancer patients with chemotherapy-induced neutropenia, and prompt prevention of FN is critical (Rivera-Salgado et al., 2018).

Granulocyte-colony stimulating factor (G-CSF) has been shown to facilitate recovery from FN in cancer patients (Qin and Ma, 2021). However, its short half-life poses challenges for patient adherence (Wang et al., 2019; Yang et al., 2015). Mecapegfilgrastim, a long-acting G-CSF developed to address this issue. Administered subcutaneously as prophylaxis 48 h after the completion of chemotherapy in each cycle. Its longer half-life allows once-per-chemotherapy-cycle administration. It is also the first G-CSF product developed in China to be recognized by the World Health Organization (WHO) (Jiangsu Hengrui Medicine Co. Ltd., 2022).

Emerging evidence suggests that mecapegfilgrastim is effective and safe for managing FN in breast cancer (Xu et al., 2020), lung cancer (Zhou et al., 2016), lymphoma (Zheng Y. et al., 2021), and other malignancies, with no unexpected adverse events (AEs) reported (Ma et al., 2021a). Studies have demonstrated that the incidence of FN is significantly lower with primary prophylaxis using mecapegfilgrastim compared to its secondary use (Ma et al., 2021). Its once-per-cycle administration offers convenience in clinical practice, providing a promising alternative for cancer patients requiring FN prevention after chemotherapy. Furthermore, mecapegfilgrastim enhances patient compliance, reduces chemotherapy-related side effects, and simplifies FN management for healthcare providers, making it a valuable addition to cancer treatment strategies (Shi et al., 2017).

Mecapegfilgrastim was initially priced at ¥ 6,800 Chinese yuan per 0.6 mL (6 mg), making it inaccessible for many cancer patients due to the high financial burden. The Chinese government implemented policies to improve access to novel anticancer medicines, such as the National Health Insurance Coverage (NHIC) policy to address this issue. Under this policy, new anticancer medicines can be included in the National Reimbursement Drug List (NRDL) through annual price negotiations between the government and pharmaceutical companies. Medicines listed in the NRDL are classified as “negotiated medicines,” Medicare beneficiaries can receive insurance reimbursement for them, improving the affordability of cancer care. For pharmaceutical companies, inclusion in the NRDL often increases sales and market share, incentivizing negotiation participation. Following the 2020 negotiations, the price of mecapegfilgrastim was reduced to ¥ 3,080 and included in the NRDL on 1 January 2020.

China’s national price negotiation process began in 2016, successfully including two targeted anticancer drugs, icotinib and gefitinib. In 2017, the Ministry of Human Resources and Social Security initiated centralized strategic negotiations, including 18 novel anticancer medicines in the NRDL, with an average price reduction of 44%. Before 2018, adjustments to the NRDL occurred infrequently, with the longest interval spanning 8 years, leaving many newly approved drugs uncovered by medical insurance. The establishment of the National Healthcare Security Administration (NHSA) in 2018 standardized the annual adjustment of the NRDL, strengthening bargaining power as the largest payer of medical expenses. By 2023, 446 drugs had been included in the NRDL through national price negotiations, with over 40% targeting tumors and chronic diseases.

Several studies have evaluated the impact of the NHIC policy on drug accessibility using national or regional procurement data. Zhang M. et al. (2021) found that 15 negotiated targeted anticancer drug costs decreased by 48.9%, while procurement volumes increased by 143% (Zhang M. et al. 2021). Cai et al. (2022a) reported that the mean availability of 17 negotiated anticancer drugs in 1,039 hospitals increased by 25.22%, with a monthly availability increase of 1.23% following the policy implementation (Cai et al., 2022a). Li et al. (2024) observed that monthly expenditures and defined daily doses of 12 innovative anticancer drugs increased by 573% and 1,400% in 1,027 hospitals between 2017 and 2019 (Li et al., 2024). Ma et al. (2022) analyzed 31 anticancer drugs in Shaanxi Province from 2019 to 2021, finding significantly higher availability of negotiated drugs compared to conventional Class B drugs, with availability influenced by regional economic development levels (Ma et al., 2022). Yang et al. (2023) noted that usage of negotiated drugs increased from 1.4%–2.1% to 2.9%–3.1% (P = 0.005) after NHIC policy implementation, although prescribing rates varied by insurance scheme and regional economic disparities (Yang et al., 2023). Xu (2024) reported a year-on-year increase in the accessibility of negotiated anticancer drugs in a tertiary hospital, with over 50% of negotiated drugs included between 2020 and 2022 (Xu, 2024).

Despite these insights, few studies have assessed the effects of health insurance policies on cancer patient outcomes using patient-level data in China. For example, one study in Fuzhou City observed an increase in the proportion of breast cancer patients receiving trastuzumab therapy after NHIC policy implementation (Diao et al., 2021). Another study in Jiangsu Province linked medical insurance coverage, age, and radiotherapy to trastuzumab use (P < 0.05), with overall survival significantly improved in the trastuzumab group (P = 0.040) (Xia et al., 2021). However, trastuzumab targets HER-2-positive patients, whereas mecapegfilgrastim is a broader-spectrum prophylactic for chemotherapy-induced neutropenia across various cancer types. There is limited evidence on how the NHIC policy impacts the utilization of novel anticancer medicines among diverse cancer populations.

This study aimed to 1) address the evidence gap regarding which cancer patient populations benefit from the NHIC policy and to what extent, 2) assess changes in mecapegfilgrastim utilization across patient subgroups before and after NHIC policy implementation, and identify factors influencing medication choices using real-world data, and 3) provide evidence for policymakers to enhance the inclusiveness and equity of the NHIC policy by reducing socioeconomic disparities in mecapegfilgrastim utilization.

## 2 Methods

### 2.1 Study design

This retrospective study used a longitudinal analysis to evaluate the utilization of the surveyed medicine from August 2018 to March 2021. The study was conducted at the Cancer Hospital of Lianyungang, a 1,307-bed tertiary cancer hospital in Jiangsu Province, Eastern China. Lianyungang City is located on the east coast of Jiangsu Province, with a population of over 4 million (in 2024) residents. The total gross domestic product of this city was CNY 436.36 billion in 2023, which represents the middle level economic development of the Northern Jiangsu region in China. The sample cancer hospitial in Lianyungang usually provide medical oncology services to all of the residents in Northern Jiangsu region, owing to it's superior specialized center for the treatment of cancers in Jiangsu Province. Multivariate statistical analyses were used to assess the determinants influencing patients’ medication choices.

Eligible cancer patients were categorized into two groups: the mecapegfilgrastim group and the non-mecapegfilgrastim group. The study period was divided into two phases: (i) the pre-NHIC policy period (August 2018 to December 2019) and (ii) the post-NHIC policy period (January 2020 to March 2021).

### 2.2 Data source and patients

Data were obtained from the electronic medical records (EMRs) within the Hospital Information System (HIS). The data included demographic information, such as age, gender, marital status, location, medical insurance status, and type of insurance system. Clinical information, including the type of cancer and tumor stage, was also collected. Additionally, treatment-related data were recorded, such as whether the patient received adjuvant therapeutic drugs and whether mecapegfilgrastim was administered.

Patients were included if they met the following criteria: age greater than 18 years and a pathologically confirmed cancer diagnosis that qualified them for mecapegfilgrastim treatment. According to the guidelines for the standardized management of tumor chemoradiotherapy-related neutropenia published by the Chinese Society of Clinical Oncology, mecapegfilgrastim is recommended as a novel adjunctive chemoradiotherapy medicine in specific cases. For primary prophylaxis, it is indicated when the incidence of FN is 20% or higher. For secondary prophylaxis, it is recommended when the incidence of FN ranges from 10% to 20% and the patient meets one or more of the following conditions: ①aged over 65 years, ②receiving full-dose chemotherapy, ③radiotherapy or chemotherapy ≥1, ④ANC <2 × 10^9^/L, suffering from persistent neutropenia or bone marrow infiltration, ⑤undergoing recent surgery or presenting with an open wound, ⑥or having abnormal liver or kidney function (bilirubin >2.0 mg/dL or creatinine clearance <50 mL/min). Additional qualifying conditions include a prior occurrence of FN, malignant hemolymphoid diseases, chronic immunosuppression (e.g., HIV infection), and poor nutritional or physical status (Qin and Ma, 2021).

Patients younger than 18 years, the incidence of FN is lower than 10% and no myelosuppression after each chemotherapy or otherwise deemed ineligible by their treating physicians were excluded.

### 2.3 Data outcome variable

The number and proportion of patients who initiated mecapegfilgrastim treatment were analyzed across different demographic characteristics, cancer types, tumor stages, and time segments relative to the implementation of the NHIC policy. Mecapegfilgrastim utilization was calculated as the proportion of eligible patients using the medication each month during the study period. Specifically, the proportion of mecapegfilgrastim usage was defined as the number of patients receiving mecapegfilgrastim divided by the total number of patients included in the study during each period, multiplied by 100%. Monthly utilization was calculated similarly, as the number of patients receiving mecapegfilgrastim divided by the total number of patients included each month multiplied by 100%.

### 2.4 Data analysis

Descriptive analyses were conducted to calculate the proportion of patients initiating mecapegfilgrastim treatment, stratified by demographic characteristics, medical insurance status, cancer type, tumor stage, and NHIC policy intervention subgroups. Chi-square tests were used to compare the distributions of patients receiving mecapegfilgrastim across these subgroups, highlighting differences in utilization patterns. A binary logistic regression model was applied to identify factors associated with mecapegfilgrastim use. The dependent variable was whether the patient used mecapegfilgrastim (1 = use, 0 = no use). Backward elimination was used to refine the model, and results were expressed as odds ratios (OR) with 95% confidence intervals (CI).

To evaluate the impact of the NHIC policy on mecapegfilgrastim utilization, an interrupted time-series (ITS) regression analysis was performed. This method assessed changes in monthly utilization over 32 months from August 2018 to March 2021, divided into pre-policy (August 2018 to December 2019) and post-policy (January 2020 to March 2021) phases. The ITS model examined three key metrics: (i) the slope of monthly utilization from August 2018 to December 2019, (ii) level changes associated with the NHIC policy implementation, and (iii) the slope of monthly utilization from January 2020 to March 2021. ITS is a robust method for evaluating public health interventions, allowing a detailed assessment of their longitudinal impact. Durbin-Watson test was employed to test autocorrelation of data. All statistical analyses were performed using Stata 15.1, with a significance threshold of P < 0.05.

## 3 Results

### 3.1 Chi-square test of the factors associated with mecapegfilgrastim usage

A total of 3,041 cancer patients were included, of whom 608 received mecapegfilgrastim, while 2,433 did not. The average proportion of patients receiving mecapegfilgrastim increased significantly from 8.17% before the implementation of the NHIC policy to 36.05% afterward (P < 0.001).

Chi-square test analysis revealed significant differences in demographic characteristics between patients who received mecapegfilgrastim and those who did not. The results in Table 1 indicate that factors such as age, gender, location, type of cancer, and tumor stage were significantly associated with mecapegfilgrastim utilization (P < 0.05). However, no significant differences were observed between the two groups regarding marital status or type of insurance program.

**TABLE 1:** Chi-square test of the factors associated with the use of mecapegfilgrastim.

Parameter		With mecapegfilgrastim (n = 608)	Without mecapegfilgrastim (n = 2,433)	Proportion (%)	χ^2^	*P*
Age	<50	95	296	24.30	20.065	0.000
	50 ∼ 59	169	594	22.15		
	60 ∼ 74	281	1,132	19.89		
	≥75	63	411	13.29		
Gender	Male	272	1,246	17.92	8.159	0.004
	Female	336	1,187	22.06		
Marital status	Unmarried	8	40	16.67	0.337	0.561
	Married	600	2,393	20.05		
Locality	Urban	295	1,320	18.27	43.307	0.000
	Rural	313	1,113	21.95		
Type of insurance program	UEBMI	230	855	21.20	4.147	0.126
	URRBMI	369	1,512	19.62		
	Uninsurance	9	66	12.00		
Type of cancer	Lung	126	564	18.26	30.809	0.000
	Breast	148	376	28.24		
	Colorectal	63	245	20.45		
	Esophagus	31	138	18.34		
	Stomach	60	340	15.00		
	Others	180	770	18.95		
Tumor Stage	I	63	215	22.66	28.448	0.000
	II	154	501	23.51		
	III	154	526	22.65		
	IV	152	629	19.46		
	NA	85	562	13.14		
Implementation of the NHIC policy	After	465	825	36.05	360.931	0.000
	Before	143	1,608	8.17		

Note: UEBMI, urban employee basic medical insurance; URRMBI, Urban-rural Residents Basic Medical Insurance.

NA, patients’ cancer stage was not available.

### 3.2 Binary logistic regression analysis

Binary logistic regression analysis was conducted to identify factors associated with using mecapegfilgrastim, and the results are presented in Tables 2, 3. Table 2 highlights the overall factors influencing mecapegfilgrastim usage. After adjusting for other variables, the likelihood of patients receiving mecapegfilgrastim increased 6.321-fold following the implementation of the NHIC policy (OR = 6.321, 95% CI: 5.124-7.798, P < 0.001). Compared to patients aged 75 years or older, those aged 50-59 were more likely to use mecapegfilgrastim (OR = 1.437, 95% CI: 1.007-2.050, P = 0.046).

**TABLE 2:** The binary logistic regression analysis results of the associated factors of patients who received mecapegfilgrastim.

Parameter		P	OR	95% CI	
Age (Ref: >75)	<50	0.063	1.471	0.979	2.210
	50∼59	0.046	1.437	1.007	2.050
	60∼74	0.054	1.371	0.995	1.889
Gender (Ref: Female)	Male	0.981	1.003	0.798	1.260
Marital status (Ref: Married)	Unmarried	0.951	0.975	0.428	2.222
Locality (Ref: Rural)	Urban	0.021	0.759	0.600	0.960
Type of insurance program (Ref: Uninsurance)	UEBMI	0.103	1.888	0.878	4.059
	URRBMI	0.337	1.449	0.680	3.087
Type of cancer (Ref: Others)	LungD	0.850	0.973	0.730	1.296
	Breast	0.001	1.644	1.216	2.222
	Colorectal	0.946	0.988	0.696	1.403
	Esophagus	0.729	0.922	0.584	1.457
	Stomach	0.329	0.834	0.580	1.200
Tumor Stage (Ref:I)	II	0.037	1.465	1.023	2.099
	III	0.086	1.369	0.956	1.962
	IV	0.408	1.166	0.810	1.679
	NA	0.771	0.944	0.638	1.396
Implementation of the NHIC policy (Ref: Before)	After	0.000	6.321	5.124	7.798

**TABLE 3:** The associated factors of patients who received mecapegfilgrastim before and after the NHIC policy.

Parameter		Before the NHIC policy				After the NHIC policy			
		P	OR	95% CI		P	OR	95% CI	
Age (Ref:>75)	<50	0.084	1.959	0.914	4.197	0.282	1.309	0.802	2.135
	50∼59	0.059	1.895	0.975	3.680	0.248	1.287	0.839	1.976
	60∼74	0.046	1.861	1.011	3.424	0.355	1.199	0.817	1.760
Gender (Ref: Female)	Male	0.211	0.764	0.501	1.165	0.432	1.116	0.848	1.469
Marital status (Ref: Married)	Unmarried	0.875	0.888	0.203	3.892	0.935	1.043	0.377	2.886
Locality (Ref: Rural)	Urban	0.810	1.052	0.696	1.589	0.004	0.648	0.484	0.868
Type of insurance program (Ref: Uninsurance)	UEBMI	0.058	6.968	0.933	52.057	0.681	1.210	0.487	3.010
	URRBMI	0.139	4.540	0.611	33.745	0.937	0.965	0.392	2.373
Type of cancer (Ref: Others)	Lung	0.790	0.931	0.551	1.574	0.871	0.972	0.688	1.372
	Breast	0.181	1.416	0.851	2.355	0.004	1.725	1.186	2.508
	Colorectal	0.821	0.927	0.479	1.792	0.955	0.988	0.652	1.497
	Esophagus	0.177	0.482	0.167	1.39	0.738	1.096	0.641	1.872
	Stomach	0.591	0.830	0.421	1.637	0.396	0.828	0.535	1.281
Tumor Stage (Ref: I)	II	0.233	1.633	0.729	3.660	0.043	1.532	1.014	2.315
	III	0.136	1.854	0.823	4.179	0.230	1.285	0.854	1.934
	IV	0.453	1.379	0.595	3.193	0.545	1.135	0.753	1.712
	NA	0.141	1.862	0.815	4.256	0.127	0.696	0.437	1.109

Note: NA: patients’ cancer stage was not available.

Patients in urban areas were less likely to receive mecapegfilgrastim than those in rural areas (OR = 0.759, 95% CI: 0.600-0.960, P = 0.021). Breast cancer patients were significantly more likely to receive mecapegfilgrastim compared to patients with other types of cancer (OR = 1.644, 95% CI: 1.216-2.222, P = 0.001). Additionally, patients with tumor stage II were more likely to receive mecapegfilgrastim than those with tumor stage I (OR = 1.465, 95% CI: 1.023-2.099, P = 0.037).

To evaluate the potential impact of the NHIC policy, associated factors influencing mecapegfilgrastim usage were analyzed separately for the periods before and after the policy implementation (Table 3). Before the policy, apart from the age subgroup, no significant differences in mecapegfilgrastim usage were observed across other subgroups. Patients aged 60–74 were more likely to use mecapegfilgrastim compared to those aged 75 years or older (OR = 1.861, 95% CI: 1.011-3.424, P = 0.046). No significant differences were found among other age subgroups. After the policy intervention, patients residing in urban areas were less likely to use mecapegfilgrastim than rural residents (OR = 0.648, 95% CI: 0.484-0.868, P = 0.004). Breast cancer patients remained significantly more likely to receive mecapegfilgrastim than those with other cancers (OR = 1.725, 95% CI: 1.186-2.508, P = 0.004). Similarly, patients with tumor stage II were more likely to use mecapegfilgrastim than those with tumor stage I (OR = 1.532, 95% CI: 1.014-2.315, P = 0.043).

### 3.3 ITS analysis changes in the trend of utilization

**FIGURE 1:**
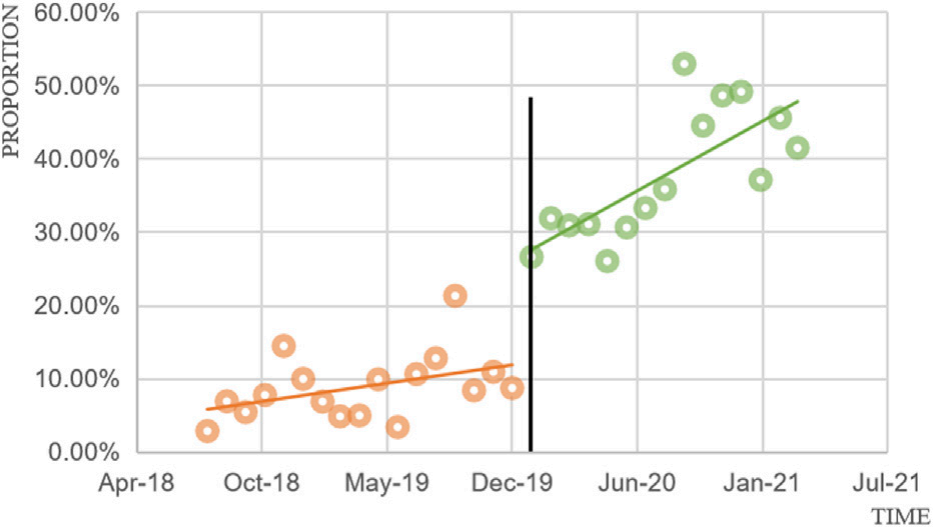
Monthly utilization of patients received mecapegfilgtastim from August 2018 to March 2021.

Figure 1 presents scatter plots depicting the observed monthly utilization of mecapegfilgrastim. The NHIC policy intervention took effect on 1 January 2020, dividing the time series into two segments: pre- and post-policy periods. According to the results in Table 4, the utilization of mecapegfilgrastim increased slightly before the NHIC policy implementation, though the increase was not statistically significant (P = 0.055). Following the policy intervention, utilization increased abruptly by 14.3% (P < 0.001) and continued to rise significantly in the post-policy period (P = 0.004), with an average monthly increase of 1.1%.

**TABLE 4:** Changes in the trend and level of utilization estimated by ITS regression analysis.

Outcome variable		Coefficient	95% CI	*P*
The utilization of mecapegfilgrastim usage	Constant	0.054	0.021–0.087	0.002
	Slope: August 2018 to December 2019	0.004	−0.000–0.008	0.055
	Chang in level: Based on NHIC policy	0.143	0.083–0.204	0.000
	Slope: January 2020 to March 2021	0.011	0.004–0.178	0.004

To validate the robustness of the findings, a sensitivity analysis was conducted within the ITS framework using April 2019 as a pseudo-intervention start date. The sensitivity test showed that the immediate effects of the NHIC policy intervention disappeared (β = −0.05, P = 0.176), confirming the stability of the observed impact of the actual intervention start date (Table 5).

**TABLE 5:** Changes in the trend and level of utilization estimated by ITS regression analysis using pseudo-start period.

Outcome variable		Coefficient	95% CI	*P*
The utilization of mecapegfilgrastim usage	Constant	0.056	0.013–0.099	0.013
	Slope: August 2018 to April 2019	0.004	−0.006–0.014	0.399
	Chang in level: Based on NHIC policy	−0.050	−0.122–0.023	0.176
	Slope: March 2019 to March 2021	0.016	0.005–0.026	0.004

## 4 Discussion

We conducted a retrospective analysis to evaluate the effects of implementing the NHIC policy for novel anticancer medicines in China. The city of Liangyungang in China is chosen as the representative city in this evaluation. A total of 3,041 cancer patients were included in this study. By using real-world data, the impact of NHIC policy on the usage of mecapegfilgrastim could be discovered in the city. This study presents changes in the utilization of mecapegfilgrastim among cancer patients with varying characteristics and identifies factors influencing patients’ medication choices based on real-world, patient-level data.

### 4.1 The implementation of the NHIC policy enhanced the mecapegfilgrastim utilization

The proportion of patients receiving mecapegfilgrastim increased significantly from 8.17% before the implementation of the NHIC policy to 36.05% afterward, indicating that a substantial number of cancer patients benefited from the policy. This shift suggests that more patients opted for novel anticancer drugs following the implementation of the NHIC policy. Binary logistic regression analysis further supported this perspective, showing that the likelihood of patients receiving mecapegfilgrastim increased by 6.321-fold after the policy was enacted. Additionally, ITS analysis revealed a significant rise in mecapegfilgrastim utilization immediately following the NHIC policy intervention (β = 0.143, P < 0.001) and an upward trend in usage after that (β = 0.011, P = 0.004). Compared to August 2018 to December 2019, the average utilization of mecapegfilgrastim increased by 28.92% between January 2020 and March 2021.

These findings align with conclusions from other studies. Liu et al. (2023) and Shang et al. (2023) examined the effects of the NHIC policy on using lenvatinib, trastuzumab, and rituximab and reported significant increases in their utilization following policy implementation. Fang et al. (2020) and Zhang Y. et al. (2021) also observed that the NHIC policy led to notable increases in the procurement volumes of novel anticancer medicines while temporarily containing overall spending. These studies highlight the NHIC policy’s role in enhancing the utilization and affordability of novel anticancer drugs, benefiting patients (Zhang Y. et al., 2021; Liu et al., 2023; Shang et al., 2023; Fang et al., 2020).

The high cost of novel anticancer medicines remains a significant barrier to widespread adoption, as it limits patient access to breakthrough therapies (Franzen et al., 2022). The NHIC policy aims to improve affordability without undermining pharmaceutical research and development. This improvement in affordability was primarily achieved through price reductions and lower out-of-pocket expenses following national price negotiations that incorporated these medicines into NRDL at acceptable price levels.

Mecapegfilgrastim is an effective and safe treatment for patients with non-myeloid malignancies receiving myelosuppressive chemotherapy (Al-Salama et al., 2019). Before the NHIC policy, mecapegfilgrastim was limited (8.17%) due to its higher cost than recombinant human granulocyte colony-stimulating factor (rhG-CSF). After the policy implementation, the price of mecapegfilgrastim was reduced to ¥ 3,080 yuan through national price negotiations, and with health insurance reimbursement, it became more affordable for patients. Despite these improvements, the utilization of mecapegfilgrastim remained relatively low. According to oncologists in the study hospital, controlling medical costs posed a barrier to the widespread adoption of novel anticancer medicines. Medical costs are a key performance evaluation metric for public hospitals, and exceeding health budgets often results in hospitals covering excess expenses. Consequently, doctors were less inclined to prescribe mecapegfilgrastim due to its high cost and associated insurance payments.

Adjustments to the reimbursement policy are necessary to ensure the effective implementation of the NHIC policy and address the low availability of mecapegfilgrastim in hospitals. For example, separating the cost of novel anticancer medicines from general cancer treatment in reimbursement policies could alleviate financial barriers. Additionally, to improve the accessibility of these medicines, the government implemented a “dual-channel” management policy in May 2021, enabling patients to obtain negotiated anticancer medicines from both community pharmacies and hospitals.

### 4.2 Demographic characteristics of patients associated with mecapegfilgrastim usage

Age was a significant factor influencing patients’ choice to use mecapegfilgrastim in this study, with younger patients being more likely to receive the medication. The primary barrier for older patients is its high cost. Each chemotherapy cycle with mecapegfilgrastim costs ¥ 6,800 yuan, which is more expensive than treatments using rhG-CSF. This financial burden significantly impacts medication choices among cancer patients. Additionally, older patients often have more chronic diseases and poorer health status, which increases their overall medical costs even with reimbursement. Another contributing factor may be that older individuals, especially retired, tend to be more price-sensitive. After implementing the NHIC policy, there was no statistically significant difference in the likelihood of mecapegfilgrastim usage among age groups, suggesting that the policy effectively benefited patients of all ages. Similar results were observed by Diao et al. (2021), who reported that insurance coverage benefited age groups with higher breast cancer incidence risks. However, their study found that patients over 60 years old benefited less than younger patients, potentially due to differences in the type of insurance program and age-related factors (Diao et al., 2021).

This study also found that patients covered by Urban Employee Basic Medical Insurance (UEBMI) and Urban-Rural Residents Basic Medical Insurance (URRBMI) were more likely to use mecapegfilgrastim than uninsured patients. However, the differences were not statistically significant. Since the 2009 healthcare reform, China has established a universal health insurance system known as Social Basic Medical Insurance (SBMI), which consists of two subsystems: UEBMI for employees and URRBMI for urban and rural residents. These systems differ in average premiums and reimbursement rates. According to the National Healthcare Security Administration, the SBMI system now covers more than 1.35 billion people, with a coverage rate exceeding 95% (National Healthcare Security Administration, 2022). Liu et al. (2023) similarly found that, compared to uninsured patients, the probability of lenvatinib use was lower among those covered by URRBMI, while no significant difference was observed for patients covered by UEBMI (Liu et al., 2023). Cross-regional insurance reimbursement policie and income disparities between insurance types may explain this discrepancy. In this study, only 75 patients (2.5%) were uninsured, some residing outside Lianyungang City or even outside Jiangsu Province. These patients may have opted for self-funded chemotherapy and sought reimbursement in their insured regions.

Contrary to these findings, Yu-Wen et al. (2014) reported that patients covered by UEBMI were more likely to select rituximab therapy than those insured under URRBMI (Yu-Wen et al., 2014). This difference may arise from disparities in claimable benefits, drug coverage, and medical examinations between UEBMI and URRBMI. However, in this study city, the reimbursement ratio for chemotherapy of malignant tumors is the same (90%) for UEBMI and URRBMI beneficiaries. This may explain the lack of significant differences in mecapegfilgrastim usage.

Interestingly, rural patients in this study were more likely to use mecapegfilgrastim than urban patients, a finding that contradicts the conclusions of Diao et al. (2021). Several factors may explain this discrepancy. First, the outbreak of COVID-19 in 2020 made hospital visits more difficult due to societal lockdowns in China. Rural patients faced additional challenges accessing hospitals, typically located in urban areas. Mecapegfilgrastim, with its once-per-cycle administration, offered a more convenient alternative for rural patients undergoing chemotherapy. Second, chemoradiotherapy often requires rural patients to travel between their homes in the countryside and urban hospitals every 7 days. This frequent travel can lead to fatigue, poor appetite, muscle soreness, and other adverse effects, severely impacting their quality of life during treatment (Cai et al., 2022). Third, previous studies have demonstrated that mecapegfilgrastim improves drug compliance among rural cancer patients by reducing their treatment burden. Cai et al. (2022b) found that the average cost of neutropenia prevention with mecapegfilgrastim for rural breast cancer patients was significantly lower compared to rhG-CSF after the inclusion of mecapegfilgrastim in the commercial medical insurance drug list (Cai et al., 2022b).

### 4.3 Clinical characteristics associated with mecapegfilgrastim usage

The results showed that breast cancer patients were more likely to receive mecapegfilgrastim compared to patients with other cancer types, likely due to the chemotherapy regimens commonly used in breast cancer treatment. Chemotherapy can significantly prolong survival in breast cancer patients, with taxane-plus-anthracycline combinations recommended as first-line neoadjuvant and advanced chemotherapy in China (Professional committee of breast cancer, 2019). However, these regimens are associated with higher toxicity and severe neutropenia (Zhang et al., 2018). Additionally, the incidence of myelosuppression is higher in advanced breast cancer compared to other cancers undergoing chemotherapy (Zhang, 2014). Studies have found that FN and infections are more frequent among patients receiving chemotherapy regimens such as docetaxel plus doxorubicin (AT), doxorubicin plus cyclophosphamide (AC), or docetaxel plus cyclophosphamide (TC) for breast cancer (Yuan et al., 2017; Do et al., 2015). A clinical trial evaluating the combination of docetaxel and epirubicin reported that 80% of breast cancer patients with stage III disease experienced grade 4 neutropenia, with one-third developing FN (Fisusi and Akala, 2019).

Furthermore, patients with stage II tumors were more likely to use mecapegfilgrastim than those with stage I tumors. Studies on various cancers, including lung, non-Hodgkin lymphoma, breast, and ovarian cancers, have consistently identified higher tumor stages as significant predictors of FN (Hosmer et al., 2011; Salar et al., 2012; Moreau et al., 2009; Sharma et al., 2006). In this study, Breast cancer accounted for the highest proportion of 32.67% in stage II cancer patients than Stages III and IV. FN was more likely to occur in breast cancer undergoing chemotherapy, and mecapegfilgrastim was more likely to be selected.

Implementing the NHIC policy widened the gap in mecapegfilgrastim usage across different cancer types and tumor stages. Mecapegfilgrastim was only recently approved in May 2018, and oncologists and patients initially favored short-acting G-CSF due to familiarity. Drug substitutability is another key factor influencing mecapegfilgrastim adoption. Before its approval, several rhG-CSF drugs, including first-generation PEG-rhG-CSF drugs like Neupogen and Neulasta, were widely used. Studies have shown no significant differences between PEG-rhG-CSF and daily rhG-CSF in terms of white blood cell (WBC) or ANC recovery times and hospitalization durations (Liu et al., 2019; Xie et al., 2018). Over time, the clinical application of mecapegfilgrastim has demonstrated its safety, efficacy, and cost-effectiveness, likely contributing to its increased usage.

## 5 Study limitations

This study has several limitations. First, it is a single-center, observational, and uncontrolled study with data collected from a regional oncology medical center in Northern Jiangsu Province. The relatively small sample size limits the generalizability of the findings. Future studies should include larger sample sizes from more representative hospitals nationwide to reduce selection bias and validate these results. Second, the data extracted from HIS lacked detailed social characteristics, such as patients’ disposable income levels and education status.

Further studies incorporating telephone follow-ups are necessary to investigate the relationships between patients’ financial burdens, education levels, and willingness to pay for novel anticancer medicines. Third, this study focused on patient characteristics in analyzing medication choices without considering physician preferences. Not all cancer diagnoses and treatments in China strictly follow national guidelines, and physician preference may significantly influence the utilization of novel anticancer drugs. Future research should examine this aspect by conducting qualitative interviews with physicians and patients to extend the findings of this study. Fourth, only the impact of the NHIC policy on mecapegfilgrastim utilization had been analysed. The expenditures of the individual patients could not been inculdedin this study. Lastly, this study’s retrospective design limits its ability to infer causality. Future studies should adopt prospective designs with larger, more diverse patient populations and include simultaneous control groups to provide more robust evidence.

## 6 Conclusion

Various factors, including patient age, geographic location, cancer type, tumor stage, and the NHIC policy intervention, influenced mecapegfilgrastim. The average proportion of mecapegfilgrastim usage increased notably following the implementation of the NHIC policy, indicating that more patients were able or willing to access this medication due to improved affordability and policy support. The significant rise in mecapegfilgrastim utilization after the NHIC policy intervention highlights its effectiveness in enhancing access to novel anticancer therapies. It demonstrates that cancer patients have benefited substantially from the policy. Nevertheless, inequities in mecapegfilgrastim usage persist among different patient subgroups. To address these disparities, the government should implement targeted measures to reduce social and economic inequality in access to novel anticancer medicines. Potential strategies include increasing the insurance reimbursement cap and decoupling the cost of expensive novel anticancer drugs from hospital medical insurance budgets. These actions could help ensure equitable access to advanced cancer treatments for all patients.

## Data Availability

The raw data supporting the conclusions of this article will be made available by the authors, without undue reservation.
